# Factors Associated With the Emergence and Spread of Visceral Leishmaniasis in the State of São Paulo, Brazil

**DOI:** 10.1111/zph.70052

**Published:** 2026-03-09

**Authors:** Vera Lucia Fonseca de Camargo‐Neves, Guilherme Loureiro Werneck

**Affiliations:** ^1^ Grupo Técnico de Epidemiologia Instituto Pasteur, Secretaria de Estado de Saúde de São Paulo São Paulo Brazil; ^2^ Departamento de Epidemiologia da Faculdade de Saúde Pública da Universidade de São Paulo São Paulo Brazil; ^3^ Instituto de Medicina Social Universidade do Estado do Rio de Janeiro ‐ UERJ Rio de Janeiro Brazil; ^4^ Instituto de Estudos Em Saúde Coletiva Universidade Federal do Rio de Janeiro – UFRJ, Cidade Universitária Rio de Janeiro Brazil

**Keywords:** climate, leishmaniasis, multilevel model, risk factors, socioeconomic, urbanisation

## Abstract

**Introduction:**

Visceral leishmaniasis (VL), once considered a rural disease in Brazil, has progressively urbanised, particularly in the state of São Paulo (SSP), where the first urban cases emerged after the detection of the vector *Lutzomyia longipalpis* in 1997.

**Methods:**

This study explores the environmental, climatic, socioeconomic, and infrastructural factors associated with the expansion and establishment of VL in urban areas of SSP. For the statistical analysis, two types of models were developed. First, for all municipalities (with or without VL), the logistic regression model was used. Second, for the municipalities in which VL occurred between 1999 and 2012, a multilevel logistic regression model was used, accounting for repeated measurements (VL occurrence or non‐occurrence in each municipality) over time. For both models, the associations between exposure variables and the outcome are expressed as odds ratios (OR) and their respective 95% CIs.

**Results:**

We found that the presence of railways, highways, and sugarcane cultivation areas, as well as high annual temperatures and evapotranspiration rates, were associated with VL occurrence. Conversely, lower VL rates were associated with higher rainfall, higher altitude, and more rainy days.

**Conclusions:**

We conclude that unplanned urbanisation, poverty, population mobility, and climate change have facilitated the spread of the disease in São Paulo. The results highlight the need for integrated surveillance and control strategies that consider the socioenvironmental determinants to prevent further expansion of VL to other vulnerable urban areas of São Paulo State and bordering states.

## Summary

Determinants of the emergence and spread of zoonotic visceral leishmaniasis (VL) in Brazil involve multiple layers of factors, from macro socioenvironmental and climate variables to local community, family, and individual features. Most studies on the determinants of VL focus on the factors associated with disease occurrence but do not consider the gradual process of disease spread in space and time. Here, we employed the framework of multilevel models for repeated measures, considering time‐varying covariates, to assess the socioeconomic and environmental factors involved in the spatial and temporal dynamics of VL in the state of São Paulo, Brazil. Our study highlights that the interconnection between major contextual socioeconomic factors, such as the presence of railways, highways, and sugarcane cultivation areas, and climate variables such as high annual temperatures and evapotranspiration rates, influenced disease emergence and spread in the state of São Paulo. Identifying the complex network of socioenvironmental factors that modulate the epidemiology of VL is essential for the design of prompt, effective, and targeted prevention and response strategies.

## 1 Introduction

Visceral leishmaniasis (VL) is a neglected disease and the most severe form among other clinical manifestations of leishmaniasis. Currently, after the impact of the VL elimination program in India, Nepal, and Bangladesh, data from the Global Burden of Disease (GBD) study estimated that 30,000 new cases and 5500 deaths occurred in 2021 due to VL (Zhang et al. 2025).

In the Americas, VL is caused by the Trypanosomatidae protozoan *Leishmania infantum* (sin. *L. chagasi*) and is transmitted mainly through *Lutzomyia longipalpis* (Diptera, Psychodidae). The domestic dog is considered to be the main reservoir of infection in urban environments. At the same time, marsupials and other canids, such as the fox, are responsible for the maintenance of the wildlife cycle of transmission. It is a systemic infection that is almost always fatal if not treated. VL symptoms in humans include weeks–months of high fevers, significant weight loss, splenomegaly, and hematologic alterations, especially thrombocytopenia (WHO 2010).

Similarly to what has been observed since the 1980s in Brazil, the process of VL spread in the state of São Paulo (SSP) is characterised by expansion to urban centers and association with different social and environmental features (Sevá et al. 2017; Santos et al. 2021). The determinants that lead to the establishment and maintenance of VL transmission in urban environments are varied and involve contextual and individual factors, from bioecological and socioenvironmental ones that favour the presence of the vector to individual factors related to the disease clinical manifestation and progression (Duarte et al. 2025; Werneck 2016; Werneck et al. 2007).

In the context of VL spread, among the factors that have been poorly explored is the introduction, modification, and expansion of certain types of crops, such as the large‐scale planting of sugarcane in the SSP. The expansion of sugarcane cultivation in Brazil started in the 1970s with the appearance of the National Ethanol Program—Proálcool—aiming to stimulate the production of ethanol, which still had an essential role in exports. From the 2000s onwards, production was observed to grow twofold in 4 years. This new panorama led to a reorganisation of the geographic space, leading to regional changes in the landscape and in the demographic profile. The state of São Paulo concentrates most of the mills and the largest areas of sugarcane crops. The SSP is also the largest national producer, responsible for almost 60% of Brazilian sugarcane (Silva and Silva 2012).

Several factors that lead to the emergence and establishment of VL transmission have been described, however, for the effective control of the disease, it is essential to expand the scope of these investigations and include other factors related to environmental transformations that result from the agricultural‐industrial productive model that allow for a better understanding of the dissemination of VL and the identification of vulnerable areas. In this sense, new statistical‐computational approaches, such as multilevel and spatiotemporal models, are being adopted to address complex phenomena such as VL transmission in urban environments and offer assistance for opportune control practices directed to the regions with the greatest risk for transmission (Werneck et al. 2007; Margonari et al. 2006; Oliveira et al. 2001; Camargo‐Neves et al. 2001).

This study describes the spatial and temporal evolution of visceral leishmaniasis in the state of São Paulo, considering the socioeconomic and environmental factors involved in this process.

## 2 Materials and Methods

### 2.1 Area of Study

The area of study includes the 645 municipalities of the state of São Paulo, located in the southeastern region of Brazil. According to estimates from the demographic census of the Geographic and Statistical Institute of Brazil—Instituto Brasileiro de Geografia e Estatística—IBGE—in 2010, São Paulo's population was 41,162,299, 95.6% of which is found in urban centers. Its average index of human development was 0.783. Among its economic activities, besides industry, agricultural business stands out, especially in the western region of the state.

Among its most important road networks are those that stretch out transversely through the state of São Paulo (SSP), such as Rodovia (Highway) Dom Gabriel Paulino Bueno Couto (from Jundiaí to Itu), and the longest stretch which corresponds to the Rodovia Marechal Rondon, 331.13 km in extension, from Bauru to Castilho (center‐west) at the border with the state of Mato Grosso do Sul. The Novoeste SA railroad comprises the western railway system within the Federal Railway System—Rede Ferroviária Federal S/A—RFFSA—and resumed activities in 1996. It includes the metric gauge lines of what was previously known as the Northeastern Brazil Railway—Estrada de Ferro Noroeste do Brasil—of 1905. The construction of this railway started in Bauru, SP, in the direction of Cuiabá, Mato Grosso (MT), and in 1952, it reached Corumbá on the border with Bolivia. It became a factor for development in the region and for the integration of the Brazilian railway system with Bolivia.

### 2.2 Data Source and Variables

Secondary sources of local human cases of VL, from 1999 to 2014, were used, obtained from the Information System of Notified Disease—Sistema de Informação de Agravos de Notificação—Sinan—in São Paulo (Secretaria de Estado da Saúde de São Paulo 2014). The following fields were considered: the date of symptom onset and the municipality where the infection occurred. The canine cases of VL per year of first detection, as well as the presence of the vector (per year of first detection in the municipality), were obtained from the information system of the Superintendência de Controle de Endemias—of the Health Department of São Paulo—SES/SP (SISZOO v.2 e FLEBWEB v.1).

The database with the geographic information of the municipalities (location, altitude), gross domestic product, production data, planted and harvested area of sugarcane, and the number of sugarcane mills per municipality was obtained from IBGE (2010).

The climatic database per municipality was initially obtained from the monthly data of 190 monitoring stations observed by the Integrated Center of Agro‐meteorological Information—Centro Integrado de Informações Agrometereológicas—CIIAGRO—from 1999 to 2014. Based on this information, the annual averages of the mean, maximum, and minimum temperature, vapour transpiration rate, air relative humidity rate, and rainfall were obtained. Subsequently, stations that had 15 to 18 annual mensuration for 15 to 18 consecutive years (number of years of the period under study) in 60 stations were selected. According to CIIAGRO's recommendations, a radius of 30 km surrounding a station was considered for collecting climatic data from other municipalities. Thus, a buffer of 30 km around each of the stations was created, and the municipalities included in this area were given the values referring to the baseline station for each year. Municipalities that were not within a station's coverage area were given the data of the municipality with the shortest distance among neighbouring municipalities. For this analysis, ArcGIS v. 10.3 software was used.

### 2.3 Data Analysis

To describe the evolution of VL from 1999 to 2014, choropleth maps were created from the first year of the detection of human, canine, and vector cases using ArcGIS v. 10.3 software.

For the assessment of factors related to the occurrence of VL, two analyses were done. Initially, all the municipalities of the SSP were considered (with and without human cases of VL). Subsequently, only the municipalities with VL were considered, the outcome being the occurrence of cases each year.

There were two exposure variables for the first analysis: socioenvironmental (altitude, the crossing of the Marechal Rondon Highway through the municipalities, the presence of a railway in the municipality, the presence of a sugarcane mill, the gross internal product (GIP) per capita, the area (ha) planted with sugarcane and the presence or absence of planted areas in the municipality); and climatic, for the period from 1999 to 2014 (minimum, average and annual minimum temperatures; average annual vapour transpiration rate; annual average rainfall rate; monthly average of days of rain).

For the second analysis, fixed variables were considered, that is, those that do not vary with time (altitude, the Marechal Rondon Highway crossing through the municipalities, the presence of a railway in the municipality, the presence of a sugarcane mill) and time‐varying variables: the gross internal product per capita, the area (ha) planted with sugarcane and the presence or absence of planted area in the municipality; and climatic variables (minimum, average and annual minimum temperatures; annual average vapour transpiration rate; annual average rainfall rate; and monthly average of days of rain). Since one of the time‐varying variables (Average Gross Internal Product per capita) spanned only from 1999 to 2012, to include this variable in the analysis, we decided to shorten the analysis period to 1999–2012.

For the statistical analysis of factors related to the occurrence of VL in the state, two types of models were developed:

1. For all municipalities (with or without VL), the logistic regression model was used, where the associations among the exposure variables and the outcome are expressed by odds ratios (OR) and the respective 95% confidence intervals (95% CI). The statistical analyses were performed in two stages: in the first stage, the socioenvironmental variables were analysed (excluding climatic). The variables that demonstrated an association with the occurrence of VL throughout the years in the multivariate analysis, with a significance level of *p* < 0.1, were included in the models for the subsequent analyses, including the climatic variables. In the second stage, the climatic variables (one by one and separately to avoid collinearity) were analysed, and later in multivariate models, including the socioenvironmental variables selected in the first stage.
2. For the municipalities in which VL occurred between 1999 and 2012, a multilevel logistic regression model was used in which repeated measurements are considered (the occurrence or non‐occurrence of VL cases in each municipality) throughout the years. Hence, as with a traditional logistic regression model, the associations among exposure variables and the outcome are expressed by odds ratios (OR) and the respective 95% CI. The software used for the analyses was DMSS‐SPSS—IBM v 24.0 and STATA v. 18.

## 3 Results

**FIGURE 1:**
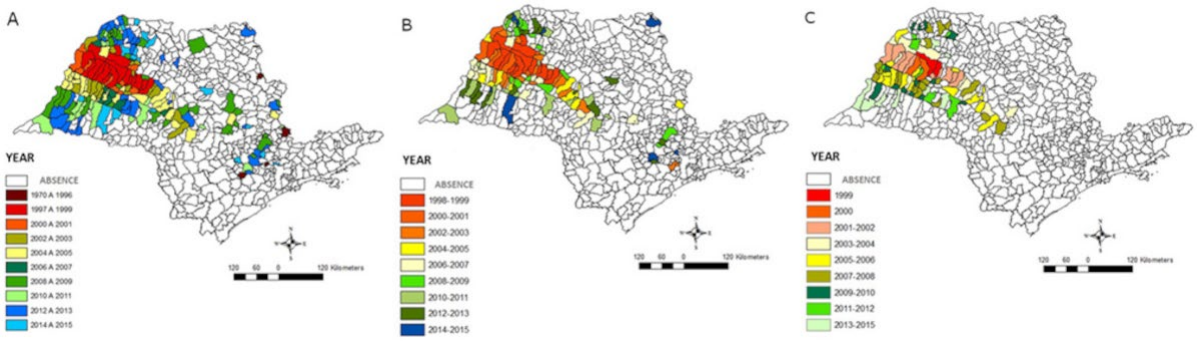
Distribution of the municipalities with the presence of *Lutzomyia longipalpis* (A), canine visceral leishmaniases (B), and human visceral leishmaniases (C), according to the first year of detection, state of São Paulo, 1970–2015.

In the state of São Paulo (SSP), from 1970 to 2015, the vector *Lutzomyia longipalpis* was registered in 184 municipalities (Figure 1a). The canine enzootic disease was registered in 126 municipalities (1998–2015) (Figure 1b), and in 88 municipalities, human cases were identified (1999–2015) (Figure 1c). In 122 municipalities, the vector and canine cases were identified, and in 86 municipalities, the vector and human cases were identified. From the first notification of an autochthonous case of VL from 1999 to 2014, the transmission of the disease in the SSP was registered in 79 municipalities, affecting both the human and canine populations.

The general incidence of human visceral leishmaniases (HVL) between 1999 and 2014 was 6.40 cases per 100,000 inhabitants, with the record of 2462 cases of VL and 204 deaths, resulting in an average mortality rate of 8.28%.

**FIGURE 2:**
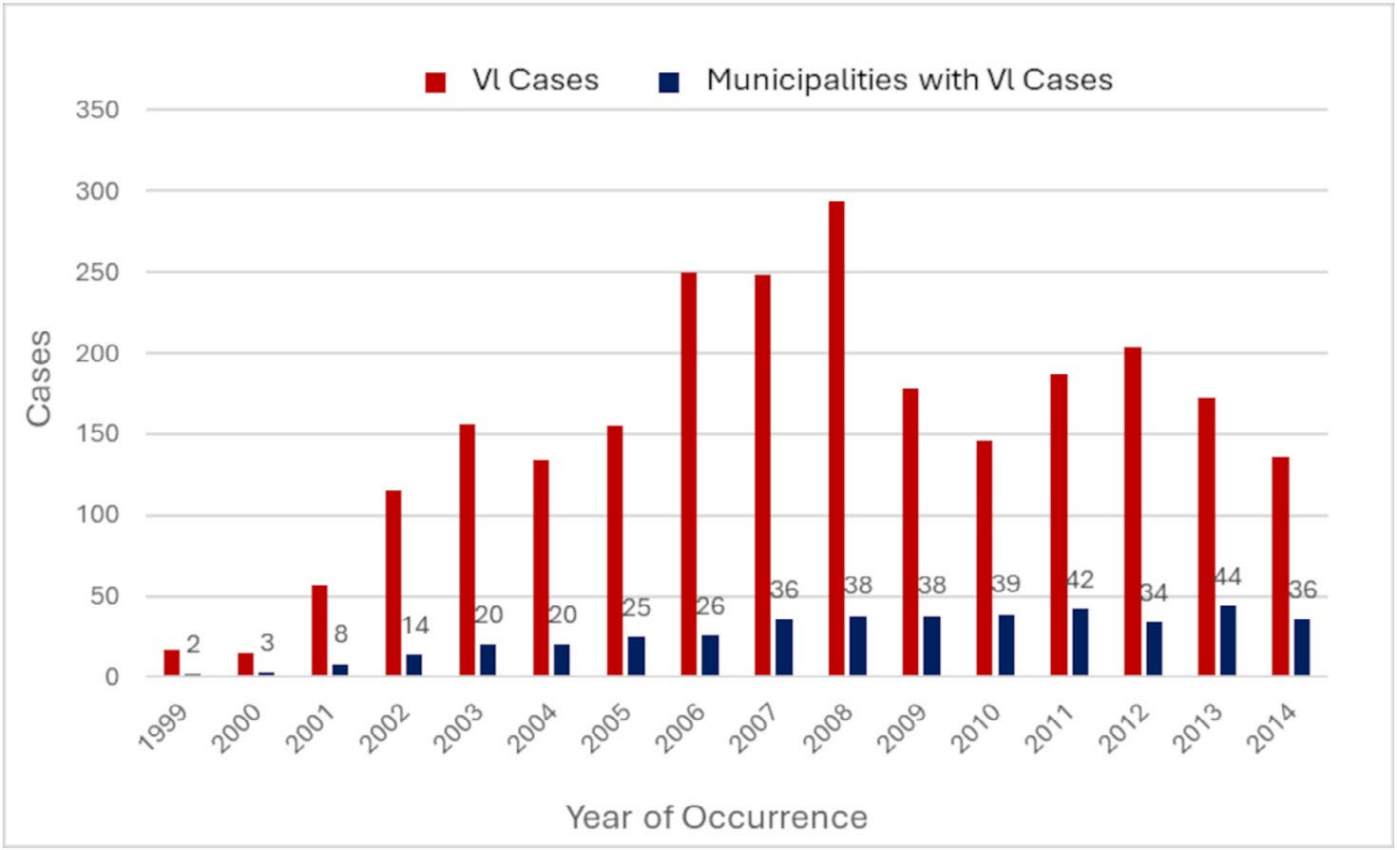
Number of human visceral leishmaniasis (HVL) cases registered and number of municipalities with cases registered, state of São Paulo, 1999–2014.

The highest incidence of the disease was registered from 2006 to 2008, with values of 10.43, 10.27, and 12.09 cases/100,000 inhabitants, respectively. In 2007, the greatest expansion of the disease was recorded, with the highest number of new municipalities with HVL cases registered, while 2012 saw the highest decline in municipalities with cases registered in relation to the previous year. Figure 2 shows the number of cases registered and the number of municipalities with cases registered per year.

Regarding the factors associated with the occurrence of VL, for the first analysis, all the municipalities of the state of São Paulo, with or without VL registered from 1999 to 2014, were selected. The outcome was the occurrence or non‐occurrence of at least one case of VL in each municipality between 1999 and 2014. Table 1 presents the results of the bivariate and multivariate analyses, including the socioenvironmental variables (excluding climatic).

**TABLE 1:** Odds ratios (OR) and 95% confidence intervals (95% CI) for cases of human visceral leishmaniasis registered, associated with socioenvironmental characteristics. Municipalities in the state of São Paulo, 1999–2014.

Socioenvironmental variables	OR*	95% CI*	*p* ^a^	OR^b^	95% CI^b^	*p* ^b^
Altitude (100 m)	0.69	0.61–0.78	< 0.001	0.66	0.57–0.77	< 0.001
Marechal Rondon Highway						
Does not cut through the center of the municipality	1.00			1.00		
Cuts through the center of the municipality	5.94	1.77–19.9	0.004	1.48	0.24–8.93	0.667
Presence of sugarcane or ethanol mill						
No	1.00			1.00		
Yes	1.08	0.63–1.86	0.761	0.80	0.41–1.54	0.511
Presence of railway or active train station						
No	1.00			1.00		
Yes	21.5	10.1–46.1	< 0.001	24.6	10.3–58.6	< 0.001
Average Gross Internal Product per capita in R$1000 (1999–2012)	0.96	0.93–1.00	0.060	0.95	0.91–0.99	0.041
Sugarcane cultivation						
No	1.00			1.00		
Yes	2.27	1.17–4.41	0.015	2.64	1.23–5.66	0.012

^a^ Not adjusted.

^b^ Mutually adjusted by the variables in the table.

Table 1 shows that (1) a higher altitude was associated with a lower chance of the municipality registering VL cases from 1999 to 2014; in other words, for every 100 m‐increase in altitude, the chance for the occurrence of VL cases was reduced by 34%; (2) the Marechal Rondon Highway cutting through the center of the municipality and the presence of sugarcane mills were not associated with the chance of the municipality registering VL cases between 1999 and 2014; (3) the presence of the railway or train station was associated with an increase of 24 times in the odds of the municipality registering VL cases between 1999 and 2014; (4) a higher GIP per capita was associated with a lower odds of VL occurrence. For every R$1000 increase in the GIP per capita, there was a 5% decrease in the chances of the municipality registering VL cases between 1999 and 2014, and (5) municipalities with sugarcane cultivations presented a 2.6 times greater chance of registering VL cases between 1999 and 2014.

Table 2 presents the results of the analyses referring to climatic variables, showing that (1) maximum, minimum, and average monthly temperatures have a direct association with VL occurrence. A 1‐degree increase in maximum, minimum, and average temperatures was associated with 2.40, 2.47, and 3.46 increases in the chances the municipality would register VL cases between 1999 and 2014; (2) the rate of vapour transpiration was associated with a higher chance of VL cases occurring. For every millimetre of increase, the chances of VL occurrences increased 16%; (3) the rainfall rate was associated with a lower chance of VL occurrence. For every millimetre of increase in rainfall, the chances of VL occurrences decreased 14%; (4) the monthly average of days of rain was associated with a decrease in the chances of the municipality registering VL cases. For every extra day of rain, the chance of VL occurrences decreased by around 50%.

**TABLE 2:** Odds ratios (OR) and 95% confidence intervals (95% CI) for the register of human visceral leishmaniasis cases associated with climatic characteristics in the municipalities of the state of São Paulo, 1999–2014.

Climatic variables	OR^a^	95% CI^a^	*p* ^a^	OR^b^	95% CI^b^	*p* ^b^
Maximum monthly temperature (1999–2014 average)	2.33	1.85–2.94	< 0.001	2.40	1.83–3.15	< 0.001
Minimum monthly temperature (1999–2014 average)	2.66	2.05–3.45	< 0.001	2.47	1.80–3.37	< 0.001
Average monthly temperature (1999–2014 average)	3.44	2.51–4.72	< 0.001	3.46	2.35–5.09	< 0.001
Average annual vapour transpiration rate (1999–2014 average)	1.15	1.11–1.19	< 0.001	1.16	1.11–1.21	< 0.001
Average annual rainfall rate (1999–2014 average)	0.86	0.82–0.89	< 0.001	0.86	0.82–0.90	< 0.001
Monthly average days of rain (1999–2014 average)	0.38	0.29–0.49	< 0.001	0.49	0.40–0.61	< 0.001

^a^ Unadjusted.

^b^ Adjusted by altitude, presence of railway or train station, average gross internal product per capita and sugar cane cultivation.

We employed a second model of analysis where only the subset of municipalities registering at least one case of HVL from 1999 to 2014 was selected. The outcome was the occurrence or non‐occurrence of at least one case of VL in each municipality from 1999 to 2014.

Table 3 shows that, for municipalities with at least one case during the period under study, (1) a higher altitude was associated with a lower chance of VL occurrence (*p* = 0.074). In other words, for every 100 m of increase in altitude, the chances for the occurrence of cases of VL was reduced 39%; (2) Marechal Rondon Highway cutting through the center of the municipality was associated with an increase in almost 9 times for the chances the municipality would register a case of human VL; (3) the existence of a sugarcane mill and cultivated area did not present any association with the occurrence of VL; (4) the existence of a railway or train station was associated with an increase of 2.6 times for the chance of the municipality registering cases of VL; (5) a higher GIP per capita was associated with a higher chance of VL occurrence. For every increase of R$1000 in the GIP per capita, there was a 12% increase in the chances of the municipality registering a case of VL.

**TABLE 3:** Odds ratio of human visceral leishmaniasis chances (OR) and 95% confidence intervals (95% CI) associated with socioenvironmental characteristics of the municipalities of the state of São Paulo. Only municipalities that registered at least one case of human visceral leishmaniasis between 1999 and 2014 were included.

Socioenvironmental variables	OR^a^	95% CI^a^	*p* ^a^	OR^b^	95% CI^b^	*p* ^b^
Altitude (100 m)	0.71	0.46–1.09	0.125	0.61	0.36–1.04	0.074
Marechal Rondon Highway						
Does not cut through the center of the municipality	1.00			1.00		
Cuts through the center of the municipality	14.1	4.38–45.3	< 0.001	8.92	1.74–45.7	0.009
Presence of a sugar or ethanol production mill						
No	1.00			1.00		
Yes	1.16	0.57–2.35	0.672	1.05	0.44–2.49	0.906
Presence of a railway or active train station						
No	1.00			1.00		
Yes	3.15	1.72–5.77	< 0.001	2.68	1.14–6.28	0.023
Gross Internal Product per capita in R$1000 (varying annually from 1999 to 2012)	1.14	1.10–1.17	< 0.001	1.12	1.08–1.16	< 0.001
Cultivated sugarcane area (varying annually from 1999 to 2012)	1.63	0.58–4.53	0.346	0.88	0.26–2.98	0.840

^a^ Unadjusted.

^b^ Mutually adjusted by the variables presented in Table 3.

In Table 4, it is observed that (1) the average annual rainfall rate was associated with a lower chance of VL occurrence. For every millimetre of increase in rainfall, the chance for the occurrence of VL cases decreased 2%; (2) the monthly average of days of rain was associated with an increase in the chances a municipality registered a case of VL. For every extra day of rain, the chance for the occurrence of VL cases increased 21%; (3) the vapour transpiration rate and temperatures—maximum, minimum, and average—did not have an association with the occurrence of VL in these municipalities.

**TABLE 4:** Odds ratio of human visceral leishmaniasis (OR) and 95% confidence interval (95% CI) associated with climatic characteristics in the municipalities in the state of São Paulo. Only municipalities that registered at least one case of human visceral leishmaniasis between 1999 and 2014 were included.

Climatic variables	OR^a^	95% CI^a^	*p* ^a^	OR^b^	95% CI^b^	*p* ^b^
Maximum monthly temperature (varying from 1999 to 2012)	0.90	0.76–1.06	0.221	0.85	0.69–1.05	0.147
Minimum monthly temperature (varying from 1999 to 2012)	0.87	0.74–1.02	0.098	1.05	0.87–1.28	0.551
Average monthly temperature (varying from 1999 to 2012)	0.83	0.67–1.01	0.073	0.94	0.73–1.20	0.633
Average annual vapour transpiration rate (varying from 1999 to 2012)	0.97	0.95–0.99	0.045	0.98	0.95–1.00	0.180
Average annual rainfall rate (varying from 1999 to 2012)	0.98	0.98–0.99	< 0.001	0.98	0.97–0.99	< 0.001
Average monthly days of rain (varying from 1999 to 2012)	1.36	1.25–1.47	< 0.001	1.21	1.11–1.33	< 0.001

^a^ Not adjusted.

^b^ Adjusted by altitude, Marechal Rondon Highway cutting through the city, the presence of a railway or train station and the gross internal product per capita.

## 4 Discussion

HVL was historically considered a rural disease in Brazil, and until 1999, the State of São Paulo (SSP) was free of HVL. It was from 1997, with the notification of *Lutzomyia longipalpis* (Lutz and Neiva 1912) in an urban area of the State, that the emergence of the disease in dogs was observed. In 1999, the first cases of human VL were recorded (Camargo‐Neves and Katz 1999).

*Lutzomyia longipalpis* showed rapid adaptation, and it appears that changes in the natural environment, due to the development of agropastoral practices, disorderly urbanisation, development of transport, and climate change were the factors associated with the establishment of VL (Quintana et al. 2020; Sevá et al. 2017; Fonseca et al. 2021).

The epidemiological pattern of the disease in urban settings, in contrast to what was known in rural areas, is the result of the evolution of a closer contact between humans and the vector. To explain the spread of VL, the indicator of occurrence of cases does not seem to be the most appropriate, since it reflects the past, but is a consequence of others that can determine its presence, among them the dependence on the adaptation and expansion of *Lu. longipalpis* in the municipalities of São Paulo (Figure 1A). The evolution of its occurrence, regardless of its density, certainly shows a possible causal relationship between the adaptation of the vector in the municipalities and the transmission of canine visceral leishmaniasis (CVL) and HVL (Figure 1B,C).

Observing the infestation process of the municipalities in the State, it seems clear that it occurred along the Marechal Rondon highway (Sevá et al. 2017; Fonseca et al. 2021), and parallel to the Novoeste railroad line, formerly the Noroeste do Brasil Railroad, which connected Bauru—SP to the Bolivian border, in the municipality of Corumbá—MS, but still today with a small number of active stations in the State for the transportation of cargo. From 2007 onwards, the expansion of the vector increased in a radial direction to the municipality of Araçatuba, to the Alta Paulista region, and to the Northwest, in 2008, in the direction to the region of São José de Rio Preto, with the expansion of the VL to these municipalities. Climatic conditions such as temperature and rainfall may be factors that determined this expansion. Thus, a new expansion scenario is to be expected for the region to the North of the State, comprising the regions of Araraquara and Ribeirão Preto, both of which have hot and dry climates. We saw that canine and human disease have been following the same direction as vector expansion in the State, that is, as the vector adapted, with the introduction of the source of infection in an urban area, transmission of VL was established.

The São Paulo State, in general, is characterised by an improvement in quality of life for the population (Akerman 1996), mainly in accessibility to health services, whatever there is another side, which is unplanned urbanisation and migration, with the concentration of an underprivileged and economically impoverished population, hence making them more vulnerable to VL. To a degree, our results regarding the dispersion of HVL corroborate those of other studies (Coura‐Vital et al. 2013; Almeida and Werneck 2014; Leça Júnior et al. 2015; Cerbino Neto et al. 2009) carried out in endemic municipalities that point out that the most significant risk of canine infection was related to locations where housing is more precarious, with dirt yards and the presence of organic material and trash, favouring the presence of phlebotomies (Lara‐Silva et al. 2015).

We analysed the occurrence of HVL using a two‐step logistic regression model, considering all municipalities of the State of São Paulo. The factor responsible for the environmental change was the expansion of sugarcane cultivation, showing over the years that the occurrence of the disease had a 2.6‐fold increased risk for municipalities in the State where there was an increase in production over the period. This factor, associated with the passage of the highway through the municipality and the presence of a railway line or railway station in an urban area, showed an increased chance of 1.48 and 24.6 times, respectively, of registering VL cases. It was alongside the railway, where the highest density of underprivileged populations was concentrated in urban areas in São Paulo State. And, the largest number of informal settlements was found alongside highways, as a result of the establishment of labour in the production and cutting of sugarcane, thereby introducing the source of infection. These factors show the vulnerability of the municipalities associated along these pathways, with low socioeconomic conditions. In this approach, it was observed that those municipalities with the highest income presented the lowest risk of VL occurrence. Sevá et al. (2017) observed a negative, but non‐significant association between socioeconomic factors and VL occurrence.

In the second step of modelling the SSP municipalities with and without notification of HVL cases, climatic factors were considered. We observed that among the climatic factors that favoured the occurrence of VL, the temperature (minimum, maximum and average), representing an increase of 2.0–3.4 times the chance of the municipality presenting a case, and the annual evapotranspiration rate, in which each millimetre increase in this rate represented a 16% greater chance of the occurrence of cases. Both are factors that directly influence the vector's evolutionary cycle, especially temperature, which is well known for its influence on vector dispersion (Gómez‐Bravo et al. 2017; Berrozpe et al. 2017; Camargo‐Neves 2004; Guzman and Tesh 2000). On the other hand, the increase in precipitation and the number of rainy days negatively influenced the occurrence of VL cases; that is, a one‐millimetre increase in the precipitation rate and for each additional rainy day, there was a decrease in the chance of VL cases occurring. The same is related to altitude; an increase of 100 m in altitude reduced the chance of cases occurring by 34%. Fonseca et al. (2021), in a study carried out in the State of São Paulo, found that the variables with the greatest impact on the seasonality of *Lu. longipalpis* were temperature, precipitation, and altitude, positively influencing its dispersion. In another study also conducted in SSP, Sevá et al. (2017) observed the influence of temperature and altitude on the occurrence of cases, but not of precipitation. Margonari et al. (2006) used spatial analysis to identify areas at risk of VL in Belo Horizonte, observed that altitudes between 780 and 880 m above sea level were associated with a higher incidence of the disease, suggesting that altitude influences the distribution of vectors and the occurrence of leishmaniasis. It is worth noting that the western region of the State of São Paulo is the hyperendemic region for VL in the State and is located between 300 and 400 m above sea level.

Still, regarding the influence of climatic conditions, the impact of El Niño, which is the most considerable interannual climatic fluctuation, affects the entire world. This phenomenon, also known as Southern Oscillation, is characterised by a large‐scale warming of the equatorial, western, and central Pacific Ocean, with events that last approximately 1 year and have an average frequency of every 3–4 years. Since 1993, several studies have demonstrated strong evidence of the epidemic risk of vector‐borne diseases in different regions worldwide (Nicholls 1993; Kovats 2000), as evident by the expansion of VL through the municipalities of São Paulo every 3–4 years (Figure 2). The influence of El Niño's oscillations on the annual fluctuations in temperatures and of these on the seasonality of VL incidence was observed in the State of Bahia, Brazil (Franke, Ziller, et al. 2002; Franke, Staubach, et al. 2002), and in other countries such as Costa Rica and Colombia (Chaves and Pascual 2006). In Sudan and Tunisia, studies have shown that VL transmission is sensitive to climatic variations and is strongly affected by rainfall changes, atmospheric pressure, temperature, and humidity (Bouattour et al. 2021; Chalghaf et al. 2016; Elnaiem et al. 2003).

In our study, considering all municipalities in the State of São Paulo, we were able to define that the areas of the State with high vulnerability of the population to VL are associated with municipalities with sugar cane production, where the railway cuts through the urban area and, along it, the areas of occupation with low socioeconomic status. For now, the VL is limited to municipalities with areas of greater social vulnerability and located in the western region of the State, associated with high annual average temperatures and low altitudes. However, long‐term climate change will undoubtedly allow the expansion of VL to areas of the State of São Paulo that are free of the disease and the vector. This is corroborated by the observation of the presence of phlebotomine sandflies in temperate zone countries, such as Belgium and Germany, which may have been influenced by global warming (Nicholls 1993) and the possibility of *L. infantum* adapting to other species of phlebotomine sandflies (Galvis‐Ovallos et al. 2017).

We verified that factors associated with the occurrence of HVL cases are related to the ecology of urban environments. Changes in land use and occupation, with the increase in agriculture, may be linked to factors that have favoured climate change, in addition to the impoverishment of the population; the ease of mobility of this population between endemic and non‐endemic areas and the lack of planning of cities as well as human occupations are related to the adaptation of the vector to environmental changes and the expansion of VL (Alirol et al. 2010; Toledo et al. 2017; Reis et al. 2019).

Also, we considered a model among municipalities with VL transmission, that is, those that presented at least one case in the period from 1999 to 2014. It was found that municipalities with higher altitudes had a 39% lower chance of occurrence of VL cases (Fonseca et al. 2021), suggesting that altitude can influence the distribution of vectors and the occurrence of leishmaniasis (Sevá et al. 2017). The planted area did not affect the outcome, since most municipalities with transmission are located in ethanol production areas.

Using the multilevel approach, it can be observed that the factors related to the occurrence of VL cases in more than 1 year were the passage of the highway through the urban area of the municipality, the presence of the railway line, and the gross domestic product. Regarding this last factor, the greater the complexity of the municipality, the greater the likelihood of cases, which was different from the model for all the municipalities in the State. Additionally, the climatic conditions, when adjusted for other factors, showed that the greatest influence on the occurrence of VL cases was associated with the monthly average of rainy days, considering only municipalities with at least one case, perhaps because it is a drier region. This finding is corroborated by a study carried out in drier regions of Tunisia on the impact of irrigation on the increase in the density of sand flies (Barhoumi et al. 2015), which impacted the occurrence of HVL. Meanwhile, a greater volume of rain was presented as a protective factor when considering all the municipalities of São Paulo. We did not observe any influence of the annual temperature on the occurrence of VL cases, probably since most of these municipalities are located in the western region of the State, in Western Planalto Paulista, which has a tropical highland climate or a tropical climate, with hot and rainy summers and drier, milder winters. Maximum temperatures can exceed 30°C in the summer, while in the winter, minimum temperatures near 10°C are not uncommon in higher‐altitude areas.

Therefore, rising temperatures and environmental degradation affect the epidemiological pattern of VL, firstly by directly influencing the vector's life cycle, reducing its life cycle, promoting population growth and greater dispersion (Camargo‐Neves 2004; El Omari et al. 2023) and, secondly, rising temperatures can affect the Leishmania cycle in phlebotomine sandflies, enhancing transmission (Hlaváčová et al. 2013).

Environmental changes, such as those evidenced in the last 20 years in the SSP, whether through the search for new work fronts, promote the displacement of people to transmission areas or even the introduction of the disease into receptive regions, due to the introduction of the infected domestic reservoir, a food source for the vector. That change in the landscape over the last few years in the State of São Paulo has led to the emergence and establishment of VL, the introduction of a source of infection and the dispersion of seropositive animals, whether through adoption programs or through the refusal of owners to hand over their infected dogs to zoonosis services, which causes owners to send their dogs to other disease‐free areas of the municipality itself or to other municipalities, leading to the establishment of a new transmission cycle. Werneck et al. (2007) observed in the municipality of Teresina, Piauí, in a study of factors associated with VL transmission, that the increasing prevalence of canine infection predicted a high incidence of human VL, as did the high prevalence of canine infection before and during the epidemic. Low socioeconomic conditions had an amplifying effect on the association between canine infection and the incidence of human VL. Although the influence of canine population density and the prevalence of CVL were not considered in the model presented in our study, we found that dog adaptation and the establishment of CVL was increased in municipalities with vector identification; the probability was 11% in the first year after vector identification, rising to 46% after the fifth year of vector identification, and the identification of the human case after the establishment of enzootic disease was 1 year.

One intriguing finding of this study is the divergent association between HVL and Gross Internal Product (GIP) per capita, and between HVL and climatic variables, across the two types of models evaluated. In the first model, higher GIP per capita was protective, but in the second analysis, limited to affected municipalities, it was a risk factor. For climatic variables, for instance, in the first model, all temperature‐related variables were significantly associated with a higher likelihood that a municipality had registered an HVL case during the study period. Nevertheless, in the second analysis, none of the temperature‐related variables were significantly associated with VL among the affected municipalities. However interesting, it is not easy to compare the results of models with different parametrizations. The first model considers GIP per capita and climatic variables as static (average) measures associated with the occurrence of at least one VL case among all municipalities of the SSP. The second model considers GIP per capita and climatic variables as time‐varying predictors of at least one VL case in each municipality during 1999–2014. Socioeconomic and climate variables measured at ecological levels might interact with other variables, including those related to the prevalence of infection in dogs, control measures, and the level of urbanisation, which are likely to vary among areas where VL has or has not been detected (Werneck et al. 2007). Further studies should be designed specifically to explore this issue.

A limitation of this study is the method used for imputing climate data for municipalities outside the 30 km buffer of a station. This method assumes climatic homogeneity over large distances, and a more robust spatial interpolation method would be more appropriate. However, the eventual misclassification produced by our methods tends to underestimate the measures of association since distant areas with varying outcomes would have similar values of the exposure variables (climate variables).

In conclusion, two models were proposed to explain the occurrence of VL considering environmental changes and climatic factors. In the first model, considering all municipalities in the State, it was found that the presence of a railway line passing through the municipality, having sugarcane plantations, the average maximum, minimum, and average annual temperatures, and the average evapotranspiration rate during the period were associated with a greater chance of occurrence of the disease. This result points to those municipalities that suffered greater landscape transformation as a result of the expansion of sugarcane cultivation. They are also characterised as municipalities with greater ease of access and a greater flow of people. It can be expected that in the SSP there will be a tendency for the expansion to stabilise, from the point of view of the adaptation of *Lutzomyia longipalpis*, temporarily limiting the disease to municipalities that have better climatic conditions for the adaptation of the vector, as long as there is no participation in the transmission cycle of *L. infantum* of other species of sand flies, already adapted to other climatic conditions. In the second model, which considered only the municipalities in the State of São Paulo with cases of VL, it was possible to show that the factors associated with the occurrence of VL were the fact that the railroad passed through the municipality (municipalities in which the poverty line was most clearly observed) and that they were located along the Marechal Rondon highway, the gross domestic product (here representing the complexity of the municipality), and the only associated climate variable was the monthly average of rainy days (as that which favours the vector cycle in the most arid region of the State of São Paulo). Therefore, local surveillance and control programs will need to mobilise resources and employ integrated multisectoral approaches, considering both the biological determinants for the establishment of transmission and the social and structural determinants of the municipality. The approaches should consider climate change resulting from agricultural and other activities that lead to environmental changes and promote changes in the landscape; changes in the physical environment resulting from urbanisation; and the social conditions of the population.

## Ethics Statement

The authors have nothing to report.

## Conflicts of Interest

The authors declare no conflicts of interest.

## Data Availability

The data that support the findings of this study are available from the corresponding author upon reasonable request.
